# Pathophysiology of Endometriosis: Insights from Immunohistochemical Analysis of Ectopic and Eutopic Tissues

**DOI:** 10.3390/ijms26135998

**Published:** 2025-06-22

**Authors:** Fahad T. Alotaibi

**Affiliations:** Department of Anatomy and Physiology, College of Medicine, Imam Mohammad Ibn Saud Islamic University (IMSIU), Riyadh 13317, Saudi Arabia; ftalotaibi@imamu.edu.sa

**Keywords:** endometriosis, immunohistochemistry, pathophysiology, inflammation

## Abstract

Endometriosis is a complex gynecological disorder characterized by the presence of endometrial-like tissue outside the uterus, leading to chronic pain and infertility. Immunohistochemistry (IHC) serves as a vital technique for elucidating the molecular and cellular differences between ectopic endometriotic tissues and eutopic endometrium. IHC reveals significant variations in the expression of inflammatory markers, adhesion molecules, and cell cycle regulators. This literature review compiles findings from various studies that assess the role of key proteins, such as leukemia inhibitory factor (LIF), cyclooxygenase-2 (COX-2), and b-cell lymphoma 2 (BCL-2), across different menstrual phases and lesion types. Notably, elevated LIF levels and increased mast cell activity in ectopic tissues underscore the inflammatory landscape of endometriosis. Additionally, altered expression of adhesion molecules like integrins and cluster of differentiation 44 (CD44) suggests modified cellular interactions, while apoptotic markers reveal a survival advantage for ectopic cells. These insights enhance our understanding of endometriosis pathophysiology.

## 1. Introduction

Endometriosis is a common gynecological condition characterized by the presence of endometrial-like tissue outside the uterus, which often leads to chronic pain and infertility. The pathophysiology of endometriosis involves complicated immune, hormonal, and inflammatory interactions that disrupt normal tissue dynamics [1,2,3]. Immunohistochemistry (IHC) has become an essential tool for investigating these alterations, especially in comparing protein expression between ectopic endometriotic tissues and eutopic endometrium. This is a narrative review of existing literature on the immunohistochemical markers associated with endometriosis. Studies were selected through comprehensive searches of databases, including Web of Science and PubMed, using keywords including “endometriosis,” “immunohistochemistry,” and “IHC”. Inclusion criteria encompassed full-text articles published in English that investigated immunohistochemical markers in human endometriosis tissues, without restrictions on study design or lesion type. This review aims to collate and interpret findings from these studies to provide an overview of the molecular landscape of endometriosis. It focuses on various immunohistochemical markers, highlighting how their expression varies across different menstrual phases and lesion types.

The first section of this review discusses IHC findings, comparing ectopic endometriosis to eutopic endometrium, emphasizing the differences in inflammatory markers and adhesion molecules. Next, we explore the variations in marker expression throughout different phases of the menstrual cycle, revealing how hormonal fluctuations affect endometriotic tissue behavior. Finally, we examine IHC findings to distinguish between deep-infiltrating and peritoneal lesions, and to elucidate the unique molecular profiles associated with each lesion type. Despite the progress in understanding the immunological aspects of endometriosis using IHC, there remains a need for a cohesive synthesis of these findings to fully elucidate the relationship between immune responses and disease manifestations. By reviewing current research, this literature review aims to bridge this gap, providing a comprehensive overview of how immunohistochemical markers relate to the pathophysiology and potential therapeutic targets of endometriosis.

## 2. Immunohistochemical Differences Between Ectopic Endometriotic Lesions and Eutopic Endometrium

IHC studies have highlighted significant variations in the expression of inflammatory markers, adhesion molecules, and cell cycle regulators, revealing the distinct biological environment of ectopic lesions (Table 1). These findings not only enhance our understanding of disease mechanisms but also facilitate potential therapeutic targets in managing endometriosis. Inflammatory and immune markers have been a focus of research over the past few decades. Elevated levels of LIF were noted in ectopic tissues, especially during the secretory phase, suggesting a role in the inflammatory milieu of endometriosis [4]. Studies have demonstrated increased mast cell presence and degranulation in ectopic tissues, highlighting their contribution to the inflammatory environment [3]. The overexpression of enzymes and metabolic proteins such as COX-2, in both eutopic and ectopic tissues, particularly during secretory phases, emphasizes its role in inflammation and prostaglandin synthesis [5]. Corticotropin-releasing hormone (CRH) and urocortin (UCN) are expressed at higher levels in ectopic tissues, suggesting a role in stress response and inflammation [6].

Adhesion molecule studies found that the alpha-5 chain was predominantly expressed in endometriotic lesions, whereas the alpha-4 chain was present in all endometriotic glands but absent in proliferative-phase endometrial glands. This indicates altered adhesion properties in endometriosis [7]. It was also found that Oct-4 (octamer-binding transcription factor 4) expression was elevated in ectopic tissues, while CD44 (a cell adhesion molecule) and E-cadherin (an epithelial adhesion molecule) were reduced, indicating altered adhesion in endometriosis [8].

Investigations of apoptotic and cell cycle regulators reveal that endometriotic lesions exhibit reduced apoptosis (lower p53) and enhanced BCL-2 expression, suggesting a survival advantage for ectopic cells [7]. Lower PIAS3 (protein inhibitor of activated STAT3) levels in endometriotic tissues suggest dysregulation of the endometriosis-associated signaling pathway [9], while higher PAI-1 (plasminogen activator inhibitor-1) expression in ectopic tissues indicates altered fibrinolytic activity [10].

Steroid receptor studies demonstrate reduced estrogen receptor expression in endometriotic tissues, with no cyclic modulation [11], affecting hormonal responsiveness. Positive immunoreactivity of PGP9.5 (protein gene product 9.5) in the endometrium of endometriosis patients, absent in controls, suggests nerve fiber involvement in endometriosis-associated pain [12].

Increased expression of heme oxygenase (HO) in red and black peritoneal lesions, with significant differences between lesion types, suggests oxidative stress involvement [13]. Overexpression of AXL (AXL receptor tyrosine kinase), SHC1 (SHC adaptor protein 1), and ACTN4 (alpha-actinin-4) in endometriotic epithelial cells indicates enhanced signaling and altered cytoskeletal dynamics [14]. Altered expression patterns of KCNA6 (potassium voltage-gated channel subfamily A member 6) and KCNK9 (potassium two-pore domain channel subfamily K member 9) in endometriotic tissues suggest disruptions in ion transport, possibly affecting cellular excitability and fluid balance [15]. Transient Receptor Potential Channels, such as TRPA1 (transient receptor potential cation channel subfamily A member 1) and TRPV1 (transient receptor potential cation channel subfamily V member 1), showed elevated expression in deep infiltrating endometriosis (DIE) samples, indicating their involvement in pain perception and sensory transduction [16]. The reviewed studies provide valuable insights into the molecular landscape of endometriosis. They highlight the significant differences in protein expression between ectopic and eutopic endometrial tissues, emphasizing the complex pathophysiology of the disease.

The results underscore the complex and heterogeneous molecular environment of ectopic endometriotic lesions, highlighting both inflammatory and adhesion-related pathways that differentiate them from the eutopic endometrium. While numerous markers, such as LIF, COX-2, and CRH, are elevated in ectopic tissues, their roles appear to be context-dependent, suggesting dynamic alterations in the disease microenvironment. Despite these findings, there remains a lack of studies directly comparing these markers across different patient populations or lesion types. This limitation affects our ability to draw definitive conclusions about their universal significance. Therefore, integrating these molecular insights with clinical data and functional studies is essential for translating these biomarkers into targeted therapies and for understanding their variability across diverse patient cohorts.

## 3. Immunohistochemical Differences Across Menstrual Phases

The menstrual cycle plays a pivotal role in the dynamic remodeling of the endometrium, orchestrated by cyclic hormonal changes. In endometriosis, these processes are disrupted, leading to aberrant tissue behavior. Immunohistochemistry experiments have provided critical insights into the variation of specific molecular markers in endometriotic and eutopic endometrial tissues across different phases of the menstrual cycle (Table 2). Understanding these variations not only advances our knowledge of the pathophysiology of endometriosis but also identifies potential phase-specific therapeutic targets and diagnostic markers.

Several studies have investigated the expression of estrogen receptor (ER) and progesterone receptor (PR) in endometriosis tissues [17,18]. It was observed that ER expression in endometrial glandular cells only slightly decreased in certain groups during the mid and late secretory phases, while PR expression showed a significant decrease at similar stages [19]. The finding aligns with the established hormonal sensitivity of endometriotic tissues. A notable finding was the inverse correlation between ER levels and apoptosis, as well as a positive association between ER levels and proliferation [20]. The proliferation index (measured using Ki-67) was highest in endometrial cells during the proliferative phase and declined through the cycle [19]. Immunostaining for melatonin receptor 1A (MR1A) and melatonin receptor 1B (MR1B) was observed in epithelial cells across all phases of the menstrual cycle [21]. During the menstrual phase, MR1A was mainly found in collapsed glands, while in the proliferative and secretory phases, it was localized primarily in the cytoplasm of glandular epithelial cells (GECs). Similarly, MR1B was present in GECs during the menstrual phase and predominantly localized within these cells during the subsequent proliferative and secretory phases. Importantly, there was no evidence of specific MR1A or MR1B immunostaining in stromal cells (SCs) at any point during the menstrual cycle [21].

The expression of integrin alpha-4 and alpha-5 beta 1 subunits was different in endometriotic tissues. The alpha-4 chain was present in all endometriotic glands throughout the menstrual cycle, but was absent in normal endometrial glands during the proliferative phase [7]. Similarly, alpha-5 beta 1 integrin was expressed in endometriotic glands but was undetectable in normal endometrial glands at any phase of the cycle [7]. These findings underline the role of integrins in the adhesion and potential invasiveness of endometriotic lesions. Other adhesion molecules, such as CD44 and E-cadherin, also demonstrated differential expression [22], with lower levels observed in the proliferative phase than in the secretory phase [8]. Additionally, their expression was reduced in eutopic endometrial tissues relative to control tissues [8].

Another key marker, BCL-2, associated with apoptosis inhibition, exhibited phase-dependent variations [20]. It was found in all phases of the menstrual cycle, but peak levels were observed during the proliferative and late secretory phases, with reduced expression in the early secretory phase [20]. This suggests that BCL-2 plays a role in promoting cell survival, possibly contributing to the persistence of endometriotic lesions.

Markers of enzymatic activity, such as xanthine oxidase and nucleotide-metabolizing enzymes like NTPDase3 (nucleoside triphosphate diphosphohydrolase 3) and NPP3 (ectonucleotide pyrophosphatase/phosphodiesterase 3), were studied for their roles in cellular metabolism and oxidative stress in endometriosis [23]. Xanthine oxidase expression was found to vary during the menstrual cycle, being lowest in the early and mid-proliferative phases, increasing in the late proliferative phase, and reaching a peak during the secretory phase [24]. However, no significant variation in xanthine oxidase expression was observed in women with endometriosis and adenomyosis, indicating a potential dysregulation of oxidative stress pathways in these conditions [24]. Similarly, NTPDase3 and NPP3 were localized to epithelial cells and exhibited maximal expression during the secretory phase, indicating their involvement in regulating cell function and energy metabolism [24].

The menstrual cycle profoundly influences the expression of many markers in both normal and endometriotic tissues. Hormonal fluctuations during the cycle drive changes in the proliferation, differentiation, adhesion, and metabolic activity of endometrial cells. The reviewed studies underscore the importance of considering the menstrual phase when analyzing IHC markers, as phase-specific differences can provide critical insights into the pathophysiology of endometriosis.

Recent metabolomic studies have further elucidated characteristic biochemical signatures in endometriosis [25]. Integrating these molecular insights could enhance our understanding of disease mechanisms and improve biomarker discovery for clinical application. Researchers have identified a biochemical profile of endometriosis, including alterations in oxidative stress pathways (e.g., elevated concentrations of β-hydroxybutyric acid), lipid metabolism, and amino acid turnover—including threonic acid and 3-hydroxybutyric acid, succinate, citrate, and lactate [25]. These metabolic disruptions often correlate with inflammatory processes and cellular survival mechanisms observed in immunohistochemical analyses, such as elevated inflammatory markers and dysregulated apoptosis regulators. Integrating metabolomic and immunohistochemical data could provide a more comprehensive molecular signature of endometriosis, facilitating the identification of novel biomarkers for early diagnosis and tailored treatment strategies.

While numerous studies have documented alterations in the expression of ER, PR, and apoptotic regulators like BCL-2 across different endometriosis tissues and menstrual phases [18,20], the findings are often inconsistent, reflecting variability in study designs, patient populations, and lesion types. For instance, while some reports suggest decreased ER and PR expression in ectopic tissues during the secretory phase, others indicate minimal or no change [17,18]. This finding underscores the complex hormonal responsiveness of endometriotic lesions. Similarly, elevated BCL-2 levels consistently point towards enhanced cell survival, yet the extent and significance vary across studies. These discrepancies highlight the necessity for standardized methodologies and comprehensive analyses that consider lesion heterogeneity and patient-specific factors. Integrating these diverse findings is essential for a better understanding of the role of hormonal and apoptotic pathways in the progression of endometriosis, which may ultimately inform targeted therapeutic strategies.

## 4. Immunohistochemical Findings in Deep Infiltrating Versus Peritoneal Endometriotic Lesions

Endometriosis manifests in various forms, including superficial peritoneal lesions (SUP), ovarian endometriomas, and deep-infiltrating endometriosis (DIE), with DIE being the most severe phenotype due to its association with chronic pain and infertility [26]. IHC is a pivotal tool for studying the expression and localization of proteins that may differentiate lesion types. By comparing deep-infiltrating endometriosis lesions with peritoneal lesions, researchers aim to uncover molecular and cellular differences that could explain variations in disease progression and responses to therapy. This review synthesizes findings from different studies that utilized IHC to investigate the biological and pathological characteristics of endometriosis lesions.

Several studies highlighted the significant role of mast cells in endometriosis lesions. A higher number of degranulating and activated mast cells was observed in deep-infiltrating endometriosis (DIE) lesions compared to peritoneal lesions [27]. Importantly, mast cells located within 25 μm of nerves were more prevalent in DIE, indicative of their role in neuroinflammation and pain mechanisms in a severe type of endometriosis [27]. Further analysis suggests that immunohistochemical findings vary across different types of endometriosis, including ovarian and DIE. For instance, higher p53 expression and reduced ER and PR levels in atypical ovarian endometriosis suggest a shift towards a more aggressive phenotype, warranting further investigation [19]. Additionally, the distinct expression patterns of proteins such as p-ERK (phosphorylated extracellular signal-regulated kinases), PIK3CA (phosphoinositide 3-kinase catalytic subunit alpha), p-AKT (phosphorylated protein kinase B), and p-mTOR (phosphorylated mammalian target of rapamycin) in ovarian endometriomas suggest activation of specific signaling pathways, which could serve as therapeutic targets [14].

The analysis of studies on the apoptotic process reveals distinct patterns of expression in endometriotic implants [28,29,30]. BCL-2 expression was prominent in the epithelial component of ectopic endometrium, with levels reaching up to 20%, particularly in cases of deep lesions [31]. In contrast, the index was lower (<8%) in mild endometriosis cases, such as superficial peritoneal lesions, indicating BCL-2’s potential role in lesion survival and severity [31]. The analysis of studies comparing DIE and peritoneal lesions reveals significant differences in the expression of inflammatory and angiogenic markers [10]. For instance, PAI-1 (plasminogen activator inhibitor-1) expression was significantly higher in GECs and SCs of DIE lesions compared to both superficial lesions and eutopic endometrium, indicating enhanced fibrin stability and angiogenesis in DIE [10]. Similarly, COX-2, a pro-inflammatory enzyme, exhibited increased expression in eutopic endometrial SCs from deep endometriosis patients compared to controls during all phases of the menstrual cycle, further highlighting the inflammatory microenvironment of DIE lesions [5].

Oct-4 expression significantly correlates with lesion severity, including cyst diameter and rASRM staging [8]. Interestingly, CD44, an adhesion molecule, demonstrates a negative correlation with the presence of peritoneal lesions, whereas E-cadherin, a cell–cell adhesion protein, is inversely correlated with DIE lesions [8]. These findings suggest that abnormal adhesion and stem cell behavior may contribute to the distinct characteristics of deep-infiltrating and peritoneal lesions. Significant differences in purinergic signaling markers were observed between lesion types [23]. CD39, a marker of ATP metabolism, was strongly expressed in the stroma of peritoneal (86%) and ovarian (59%) lesions, but was minimally detected in DIE (36%). Similarly, stromal CD73 expression was highest in peritoneal lesions (71%), followed by ovarian endometriomas (48%) and deep lesions (22%) [23]. These data suggest that purinergic signaling and stromal heterogeneity may play a role in the progression and severity of endometriotic lesions.

The role of sensory signaling in DIE is highlighted by the increased epithelial expression of TRPA1—an ion channel involved in pain perception—in DIE samples compared to controls [16,32]. This finding aligns with the increased pain symptoms commonly associated with deep-infiltrating endometriosis and underscores the contribution of sensory pathways to disease pathophysiology [33].

Consistent structural differences between stromal and epithelial components have been observed across lesion types. For instance, CD73 epithelial labeling was present across all lesion types, but its stromal expression decreased with lesion severity [23]. Additionally, recurrent correlations between protein expression and clinical parameters, such as lesion diameter and stage, emphasize the clinical relevance of these markers [23].

Synthesis of the IHC findings underscores that DIE exhibits a distinct molecular and cellular profile compared to peritoneal lesions, reflecting its more aggressive clinical behavior. Notably, the increased presence of mast cells near nerves, along with elevated neuroinflammatory markers such as TRPA1, highlights the complex neuro-immune interactions driving pain in DIE. Conversely, markers associated with apoptosis (e.g., BCL-2), adhesion (e.g., E-cadherin), and stromal purinergic signaling (e.g., CD39, CD73) reveal alterations that may facilitate lesion invasion and persistence. While individual studies identify specific biomarkers, integrating these data suggests that DIE involves coordinated dysregulation of inflammatory, apoptotic, and adhesion pathways, presenting potential therapeutic targets. Overall, this comparative analysis emphasizes the importance of considering the heterogeneous molecular landscape when developing tailored interventions for different endometriosis phenotypes.

## 5. Immunohistochemical Characterization and Comparison Between Ectopic and Non-Endometriosis Tissues

Understanding the molecular and cellular alterations in ectopic tissues compared to eutopic and non-endometriotic tissues is crucial for developing targeted therapies for endometriosis. The complex landscape of protein expression in endometriosis tissues, especially when compared to non-endometriotic controls, provides significant insights into disease pathophysiology.

The comparison of eutopic endometrial tissues from women with endometriosis and those with non-endometriosis tissues produces several key findings. Notably, several studies report decreased levels of proteins such as LIF, ARID1A (AT-rich interaction domain 1A), and HNF4A (hepatocyte nuclear factor 4 alpha) in the endometriosis tissues [4,31]. This reduction may contribute to altered endometrial function and disease progression. For instance, the loss of ARID1A, although rare, suggests aberrant signaling pathways in endometriosis [34]. Additionally, the significant reduction of CRISPLD2 protein (cysteine-rich secretory protein LDC) in the secretory phase further indicates disrupted endometrial remodeling processes critical for embryo implantation [35].

Ectopic endometrial tissues exhibit unique molecular signatures that differentiate them from non-endometriotic tissues [36,37]. Elevated expression of proteins, such as CyPA (cyclophilin A), in both ectopic and eutopic tissues suggests a common pathway driving endometriosis pathology [38]. Additionally, increased nerve fibers and altered vascularity in ectopic tissues indicate a microenvironment conducive to inflammation and pain, hallmark symptoms of endometriosis [36,39].

HDAC2 (histone deacetylase 2) exhibits significantly higher expression levels in endometriotic tissues, which may indicate altered epigenetic regulation contributing to the disease’s persistence and progression [40]. Notably, a diminished expression of PTEN (phosphatase and tensin Homolog) in a subset of patients suggests a loss of tumor suppressor function that could facilitate lesion growth [34].

The immune landscape in endometriosis is characterized by various immune cell types, including T-lymphocytes, B-lymphocytes, and macrophages [41,42,43]. The abundance of these immune cells correlates with a pro-inflammatory microenvironment, complicating the disease’s pathology. Increased expression of CD34+ vascular markers signifies increased angiogenesis, facilitating the persistence and growth of ectopic lesions [44].

Immunohistochemical analysis in endometriosis shows a pronounced presence of inflammatory cells, including CD3+ T-lymphocytes [44] and CD68+ macrophages, particularly in cases with adenomyosis [44]. This pro-inflammatory microenvironment may maintain the cycle of inflammation and tissue damage that is characteristic of endometriosis. Additionally, the expression of Ki67—a marker of cellular proliferation—was elevated, indicating increased mitotic activity in areas affected by adenomyosis [44].

Comparative studies on diabetic endometriosis reveal intriguing differences in protein expression patterns. Higher levels of ESR1 (estrogen receptor 1) and ESR2 (estrogen receptor 2) in diabetic endometriosis tissues suggest a potential link between metabolic disturbances and hormonal signaling in disease progression [45]. The differential expression of immune markers like CD68 indicates variations in inflammatory responses associated with diabetes, influencing clinical management [45].

Distinct cellular expression profiles have been highlighted in endometriotic tissues [46]. For instance, P-NR4A1 (phosphorylated nuclear receptor subfamily 4 group A member 1) expression is significantly higher in ovarian endometriotic SCs compared to endometrial SCs, indicating a potential role in ovarian endometriosis pathophysiology [47]. Furthermore, the expression of CB1 (cannabinoid receptor 1) and CB2 (cannabinoid receptor 2) receptors is intensely detected in the epithelial cells of ovarian endometriotic lesions, suggesting involvement of the endocannabinoid system in pain and inflammation modulation [48].

The studies also reveal critical insights regarding the epithelial components of endometriotic lesions. NTPDase3 expression is primarily localized to the epithelial component of endometriotic lesions, with varying intensity based on lesion type. This is suggestive of the differential roles of NTPDase3 in various endometriotic environments, influencing local tissue remodeling and inflammation [23]. Additionally, nerve growth factor (NGF) is exclusively localized to the glandular epithelium of endometriotic lesions, with strong staining in ovarian endometriotic lesions, indicating a mechanism contributing to chronic pain [49].

Another significant finding is the expression of phosphorylated Ezrin in the stroma of all endometriotic lesions. As a cytoskeletal protein implicated in cell adhesion and migration, its phosphorylation may contribute to the invasive characteristics of endometriotic tissues, emphasizing the active role of structural proteins in endometriosis pathogenesis [50].

In summary, these findings enhance our understanding of the disease’s pathophysiology and open avenues for potential therapeutic interventions, underlining the complexity of endometriosis and the need for targeted treatments.

## 6. Immunohistochemical Evaluation of Pain-Related Markers in Endometriosis

Endometriosis often leads to chronic pain conditions such as dysmenorrhea, dyspareunia, and dyschezia. The expression of ER-α in both epithelial and stromal tissues was found to be significantly higher in patients with moderate to severe dysmenorrhea [51]. Notably, nerve fiber density around endometriotic lesions correlated with pain severity, indicating a link between NGF expression and dysmenorrhea [41]. Additionally, one study highlighted that higher levels of α-SMA (alpha-smooth muscle actin) and Slit2 (slit guidance ligand 2) staining were associated with increased fibrosis and pain severity [31].

The correlation between dysmenorrhea severity and various immunohistochemical markers has been extensively explored [44,52,53]. Increased expression of PAI-1 in GECs and SCs was significantly associated with higher dysmenorrhea scores [10]. Additionally, studies found that VAS scores for dysmenorrhea were negatively correlated with lesional PR-B (progesterone receptor B) staining levels [29,44], but positively correlated with α-SMA and Slit2 staining levels, as well as with the extent of lesional fibrosis [29,44]. This indicates that lower PR-B expression may lead to increased pain, whereas higher levels of α-SMA and Slit2 may correlate with more severe disease.

COX-2 expression has been implicated in the pathophysiology of endometriosis [6,46,54]. COX-2 was significantly higher in eutopic endometrial SCs among women with severe dysmenorrhea (pain scores ≥ 7) during the early and mid-secretory phases [5]. The elevation of COX-2 suggests its involvement in inflammatory processes that could exacerbate pain. This finding highlights COX-2’s potential as a therapeutic target for pain management in endometriosis. Cyclophilin A (CyPA) expression has been shown to be correlated with pain and recurrence in endometriosis [38]. Higher glandular CyPA expression was associated with endometrioma recurrence, while stromal and vascular endothelial CyPA expression correlated with dysmenorrhea recurrence [38]. These relationships indicate that CyPA may be a significant factor in the inflammatory milieu of endometriosis, influencing both pain severity and the likelihood of recurrence.

Elevated stromal ER-A and aromatase levels were observed in lesions from patients reporting severe dyspareunia [51]. These findings suggest that hormonal influences, especially estrogen, play a critical role in modulating pain during intercourse. The expression of IL-1β in the epithelium was significantly associated with increased nerve fiber density and NGF expression [55]. Moreover, TRPV1 expression on ectopic epithelial cells and macrophages was found to correlate with dyspareunia severity [16], indicating a role for these markers in pain perception. In general, the prevalence of deep dyspareunia in endometriosis patients has been associated with specific immunohistochemical markers such as NGF and COX-2.

Research has indicated higher glandular epithelial ER-α expression in patients suffering from severe dyschezia [51]. A notable finding was the inverse correlation between dyschezia severity and Ezrin intensity [50]. Additionally, the expression of transient receptor potential ankyrin 1 (TRPA1) and TRPV1 in both epithelial and stromal tissues was positively correlated with dyschezia severity [16], further underlining the relationship between these markers and pain. Higher ER-α expression levels were noted in patients who experienced recurrence of pain after one year [51]. These findings suggest that hormonal receptors might play a significant role in predicting pain recurrence, reinforcing the importance of molecular profiling in managing endometriosis. One study explored the impact of progestin treatment on different markers and found significant differences in expression levels of proteins such as colony-stimulating factor 1 (CSF1), mammalian Ste20-like kinase 1 (MST1), matrix metalloproteinase 1 (MMP-1), and matrix metalloproteinase 3 (MMP-3) [56]. Lower levels were observed in the Dienogest-treated group, suggesting a potential therapeutic effect of progestins on pain modulation in endometriosis. The efficacy of progestin treatment in alleviating endometriosis-associated pain has been a focal point of several studies. The H-score was employed to quantify PR status, with higher scores indicating a greater presence of PR expression [57]. Results revealed that responders to progestin therapies had significantly elevated H-scores compared to nonresponders. This suggests that PR status may serve as a predictive biomarker for treatment outcomes in endometriosis [58].

The diverse array of immunohistochemical markers examined across studies underscores the complex and multifaceted nature of endometriosis-associated pain, with each marker contributing distinctively to the underlying pathophysiology. Elevated expression levels of markers such as ER-α, COX-2, and NGF consistently correlate with increased pain severity, highlighting their potential roles in the inflammatory and neurogenic pathways involved in pain modulation. Additionally, nuanced relationships have emerged, including an inverse association between PR-B expression and dysmenorrhea, and a positive correlation between fibrosis markers, such as α-SMA and Slit2, and pain intensity. These findings suggest that specific molecular signatures may serve as valuable diagnostic indicators and therapeutic targets. However, the heterogeneity among studies—pertaining to patient populations, lesion types, and experimental methodologies—limits the ability to draw definitive conclusions. Future research should prioritize comparative analyses and functional assessments to elucidate the mechanistic roles of these markers more clearly. Such efforts will be crucial in advancing personalized treatment strategies. Ultimately, integrating these immunohistochemical insights into a cohesive framework is a critical step toward translating molecular findings into clinical practice.

## 7. Conclusions

**Figure 1:**
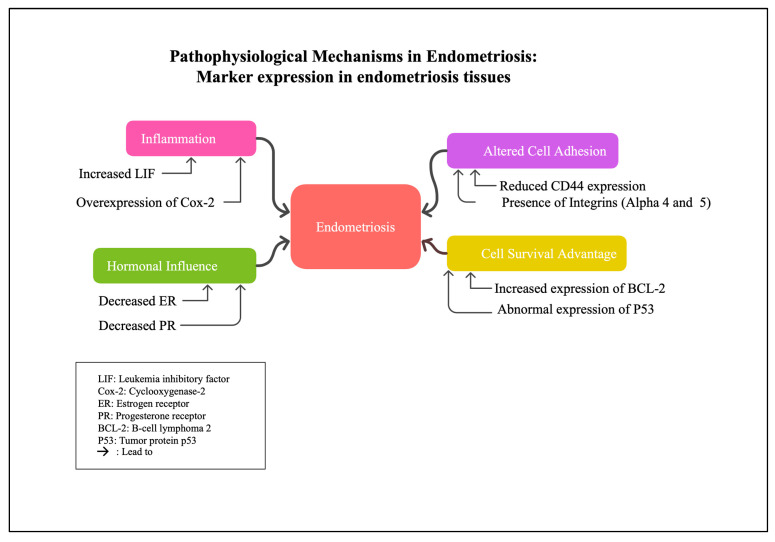
Unraveling the complex pathophysiology of endometriosis: focusing on key biomolecules and mechanisms including hormonal influences, inflammation, and cell survival.

This comprehensive review underscores the complex immunohistochemical landscape of endometriosis (Figure 1), revealing distinct molecular profiles across lesion types, menstrual phases, and clinical phenotypes. Several key patterns emerge that deepen our understanding of the disease’s pathophysiology and hold potential translational value.

First, inflammatory markers, such as LIF, COX-2, and stress-related proteins like CRH, are markedly elevated in ectopic tissues, particularly during the secretory phase. These findings suggest that hormonal fluctuations modulate the inflammatory microenvironment, potentially contributing to symptom severity and lesion persistence. The abundant presence of mast cells and oxidative stress markers (e.g., Heme Oxygenase) further emphasizes the role of inflammation and oxidative stress in lesion maintenance and pain generation.

Second, adhesion molecules—including integrins (α5 and α4 chains), CD44, and E-cadherin—demonstrate altered expression patterns, indicating disrupted cellular adhesion and increased migratory capacity of ectopic endometrial cells. Notably, the overexpression of pluripotency markers like Oct-4 and the dysregulation of apoptosis regulators—characterized by reduced p53 and increased BCL-2—indicate enhanced cellular survival and plasticity. This potentially promotes lesion establishment and resistance to cell death.

Third, variations in ion channel expression (e.g., TRPA1, TRPV1, KCNA6, KCNK9), especially in DIE, correlate with increased pain perception. This aligns molecular findings with clinical symptoms. These pain-associated markers highlight potential targets for therapeutic intervention aimed at alleviating symptoms.

Comparative analysis across lesion types reveals that deep-infiltrating lesions exhibit higher expression of pain-related channels and oxidative stress markers, whereas superficial peritoneal lesions tend to show a different inflammatory and adhesion profile. These molecular differences may explain variability in clinical presentation and treatment response.

Clinically, these insights suggest that targeted modulation of inflammatory pathways, adhesion molecules, and pain mediators could enhance management strategies. For example, anti-inflammatory agents targeting COX-2 or mast cell stabilizers, combined with therapies aimed at disrupting aberrant adhesion or neuronal sensitization, may provide personalized benefits depending on lesion type and menstrual phase.

In conclusion, integrating immunohistochemical profiles with clinical phenotypes provides a nuanced understanding of endometriosis that facilitates the development of diagnostic biomarkers and personalized therapies. Future research should focus on longitudinal studies correlating molecular profiles with disease progression and treatment response. Additionally, exploring the therapeutic potential of modulating identified pathways could help alleviate symptoms and inhibit lesion growth.

## Figures and Tables

**Table 1:** Comparison of immunohistochemical markers in ectopic vs. eutopic endometrial tissues.

IHC Marker	Expression Pattern in Ectopic Tissue	Expression Pattern in Eutopic Endometrium	Associated Clinical Features/Functional Relevance
LIF	Elevated, especially during secretory phase	Lower levels	Involved in inflammatory milieu; may contribute to implantation failure and inflammation
COX-2	Overexpressed, particularly during secretory phase	Lower	Promotes inflammation and prostaglandin synthesis, involved in pain and lesion development
CRH	Higher levels	Lower or absent	Stress response, inflammation modulation
Integrins (Alpha-5 chain)	Predominantly expressed	Lower or absent	Altered adhesion properties in endometriosis lesions
Integrins (Alpha-4 chain)	Present in all endometriotic glands	Absent in proliferative phase endometrial glands	Indicates altered cellular adhesion in lesions
Oct-4	Elevated in ectopic tissues	Lower or absent	Marker of pluripotency, linked to cellular plasticity
CD44 & E-cadherin	Reduced	Normal levels	Altered cell adhesion, increased migratory potential
p53 (apoptotic regulator)	Reduced expression	Higher in eutopic endometrium	Suggests decreased apoptosis in ectopic tissues
BCL-2	Increased	Lower	Anti-apoptotic, promotes cell survival in ectopic tissues
PIAS3	Lower levels	Higher	Dysregulation of STAT3 signaling pathway
Estrogen receptor (ER)	Lower amounts, no cyclic modulation	Higher and cyclically regulated	Hormonal responsiveness, influences proliferation
PGP9.5 (nerve fiber marker)	Positive in ectopic tissues	Absent in controls	Nerve involvement, pain perception
Heme oxygenase (HO)	Increased, varies by lesion type	Lower or absent	Oxidative stress marker
AXL, SHC1, ACTN4	Overexpressed	Lower or absent	Signaling pathways, cytoskeletal dynamics
TRPA1, TRPV1	Elevated in deep infiltrating endometriosis	Lower or absent	Pain perception, sensory transduction

**Table 2:** Immunohistochemical marker expression during menstrual cycle phases in endometriosis.

IHC Marker	Expression Pattern During Menstrual Cycle	Notes/Functional Relevance
ER	Slight decrease during mid/late secretory phases	Hormonal sensitivity, proliferation regulation
PR	Significant decrease during secretory phases	Hormonal regulation, differentiation
Ki-67 (Proliferation marker)	Highest during proliferative phase, declines thereafter	Cell proliferation status
MR1A	Present across all phases; mainly in glands	Circadian regulation, possibly linked to tissue response
MR1B	Present in glands throughout cycle	Similar to MR1A, involved in circadian signaling
Integrin alpha-4	Present in all phases	Adhesion during cycle
Integrin alpha-5	Increase during proliferative phase, decrease during secretory phases	Cell adhesion and migration
